# Supplementary material for: Spatial regulation of contractility by Neuralized and Bearded during furrow invagination in Drosophila
Source: Nat Commun. 2017 Nov 17;8:1594. doi: 10.1038/s41467-017-01482-8 (PMC5693868; doi:10.1038/s41467-017-01482-8)
Supplement: Supplementary file 1 — Supplementary Information [file 41467_2017_1482_MOESM1_ESM.pdf]

Supplementary Information

Supplementary Table

BrdDefF	GAC GCA TGA TTA TCT TTT ACG TGA C	Df(3)Brd-C1	Figure 3<br>Movies 5, 7, 8 and 11
BrdDefR	AAT GAT TCG CAG TGG AAG GCT		
EsplDefF	TGC ATT TGC CTT TCG CCT TAT	Df(3)E(spl)mδ-m6[XPd08311-RBe00084]	Figure 3
EsplDefR	AAT GAT TCG CAG TGG AAG GCT		
BrdF	ATG GCC TAC GAA ACT CTG ATG AAC ACC	Brd open reading frame	Figure 3<br>Movies 5, 7, 8 and 11
BrdR	TTA CAG TTG GAC GAG CTC GGC TTC CAA TTG C		
MBSF	CGT ATA TAA GGA GTC GGT CGT GCA GCA GGA AAA CC	Excision of the FRT-Stop cassette	Figure 7<br>Sup Figs 10 and 11<br>Movies 10 and 11
MBSR	CCG ACT CCT CCC AGC GCT TCA GCT GCT CGG CGC GC		
RhoF	CGT ATA TAA GGA GTC GGT CGT GCA GCA GGA AAA CC	Excision of the FRT-Stop cassette	Figure 6<br>Sup Fig 9<br>Movies 8 and 9
RhoR	CGG GAT AGC TCA GCG GTC GTA GTC TGT CGT AGT CC		
snaBrdF	GAG AAC GCA ACC ACC GCT CTA TAC TCG ATC CCG	Excision of the FRT-Stop cassette	Figures 1,2<br>Sup Figs 2 and 4<br>Movies 1
snaBrdR	CTC TGG CAG ACA GGA GTG GTGGTG TAG ATG GTG G		

Supplementary Table 1. List of primers used for single embryo PCR genotyping.

Supplementary Figures

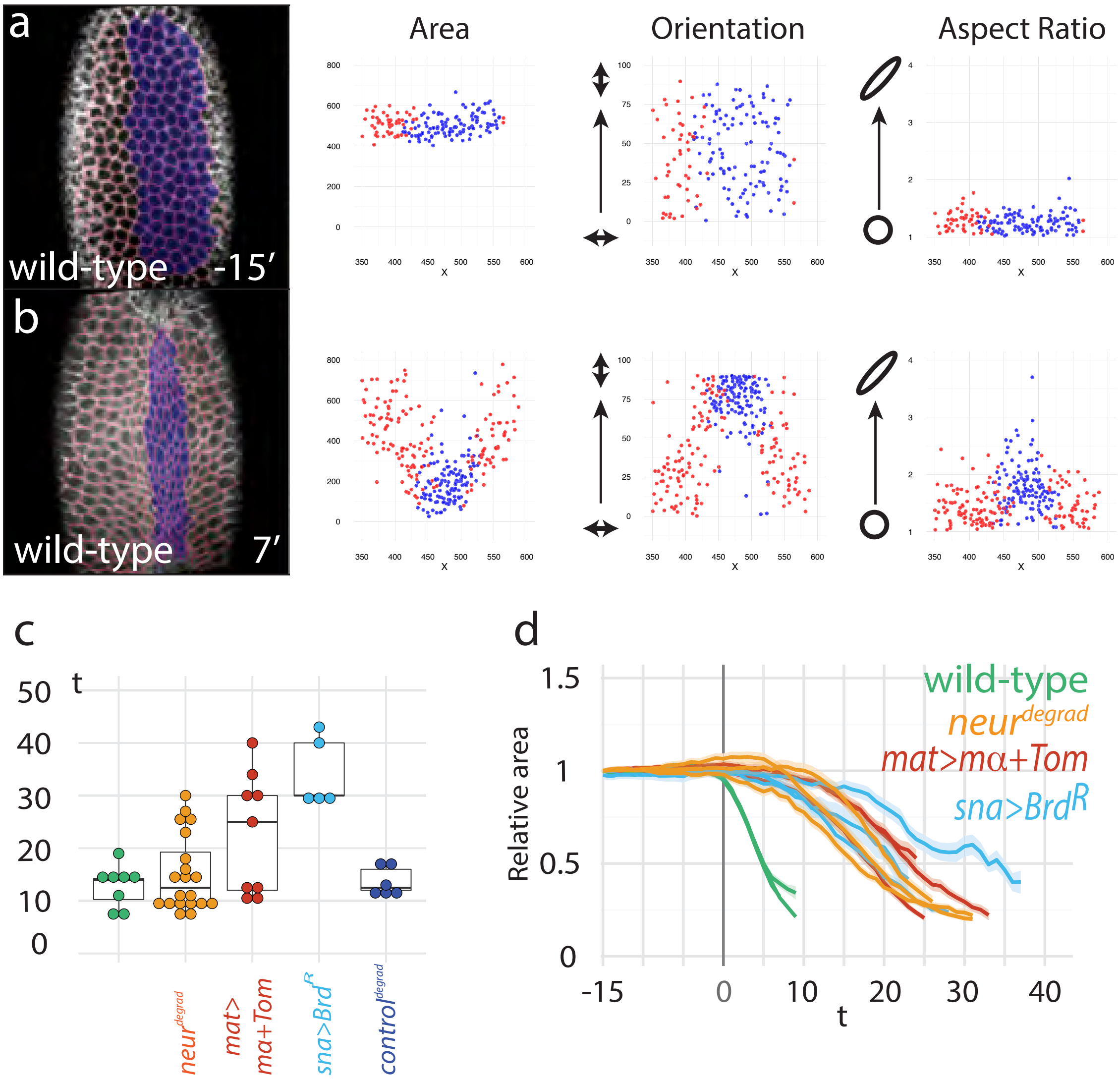

Supplementary Figure 1. *Related to Figure 1.* Ectopic Brd inhibits apical constriction

a,b) snapshots of a movie showing a wild-type embryo expressing a Gap43-Cherry (white; tracking in red) and GFP-MyoII (not shown) at t=-15 (a) and t=7 (b). Shape analysis of ventral cells showed that all cells have initially similar area, orientation and aspect ratio values but that the ~6 ventral-most cells (blue-shaded) displayed smaller area values (reflecting apical constriction) and higher anisotropy values with their long axis aligned along the anterior-posterior, i.e. furrow, axis whereas the ventral-lateral cells (red) showed heterogeneous apical area values and displayed anisotropy oriented along the DV axis.

c) Box plots of time intervals from the onset of apical constriction to furrow invagination in wild-type (n=8), *neur*^{degrad} (n =20), *mat>m*α*+Tom* (n=9), *sna>Brd*^{R} (n=5), and *control*^{degrad}

(n=6) embryos (see Methods for detailed genotypes). Invagination was significantly delayed upon depletion or inhibition of Neur. The variability in delay seen between *mat>mα+Tom* embryos likely resulted from differences in transgene copy number. Also, the variability seen between *neur^{degrad}* embryos possibly resulted from differences in zygotic genotype for the *neur* gene and age-dependent differences in deGradFP efficiency in females (see Methods). d) Kinetics of apical constriction in individual wild-type (n=3), *mat>mα+Tom* (n=3), *sna>Brd^R* (n=3) and *neur^{degrad}* (n=3) embryos. Mean surface values of constricting ventral cells (see Figure 1f) were plotted over time. Apical constriction (on average) occurred at a slower rate upon both inhibition of Neur by Brd and depletion of Neur using degradFP.

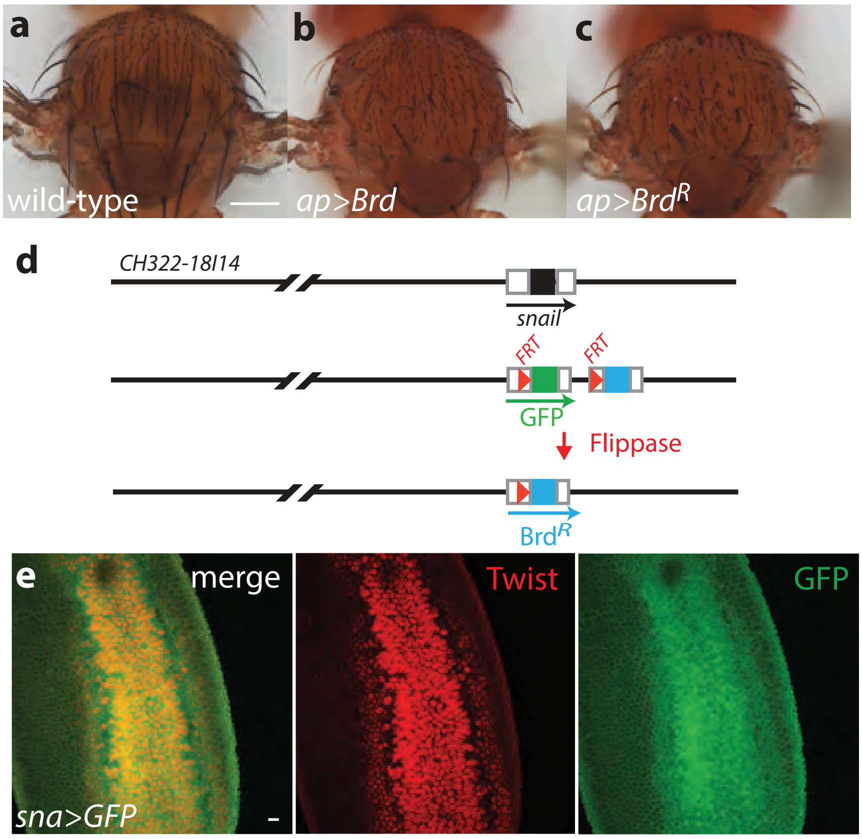

Supplementary Figure 2. *Related to Figure 1*. Conditional expression of Brd^R in the mesoderm

a-c) A mutant version of Brd, Brd^R, appeared to be more active in a gain-of-function assay. Over-expressed Brd^R appeared to antagonize the activity of Neur during bristle development more effectively than Brd (n=1 experiment). Since these two transgenes differed only by K-to-R mutations in the *Brd* gene that are predicted to prevent ubiquitin-dependent degradation, we suggest that increased levels of Brd^R results from increased stability rather than higher rate of synthesis.

d,e) Strategy to express Brd^R in all mesodermal cells. The *cis*-regulatory elements of the *snail* gene encoded within a 22 kb Bacterial Artificial Chromosome (BAC) were used to drive the expression of Brd^R in the mesoderm (d). Conditional expression was achieved using a FRT-GFP-stop-FRT cassette so that Brd^R was expressed in the mesoderm only upon FLP-mediated excision of this stop cassette. Flp-mediated excision of the FRT-GFP-stop-FRT cassette produced a *sna>Brd^R* transgene expressing Brd^R (d). In the absence of recombination, GFP (green) was expressed in all mesodermal cells (Twist, red; n=2 experiments). Single embryo PCR was used to genotype embryos carrying a recombined transgene, i.e. expressing Brd^R and not GFP. Embryo genotyping was performed following live imaging (see Methods).

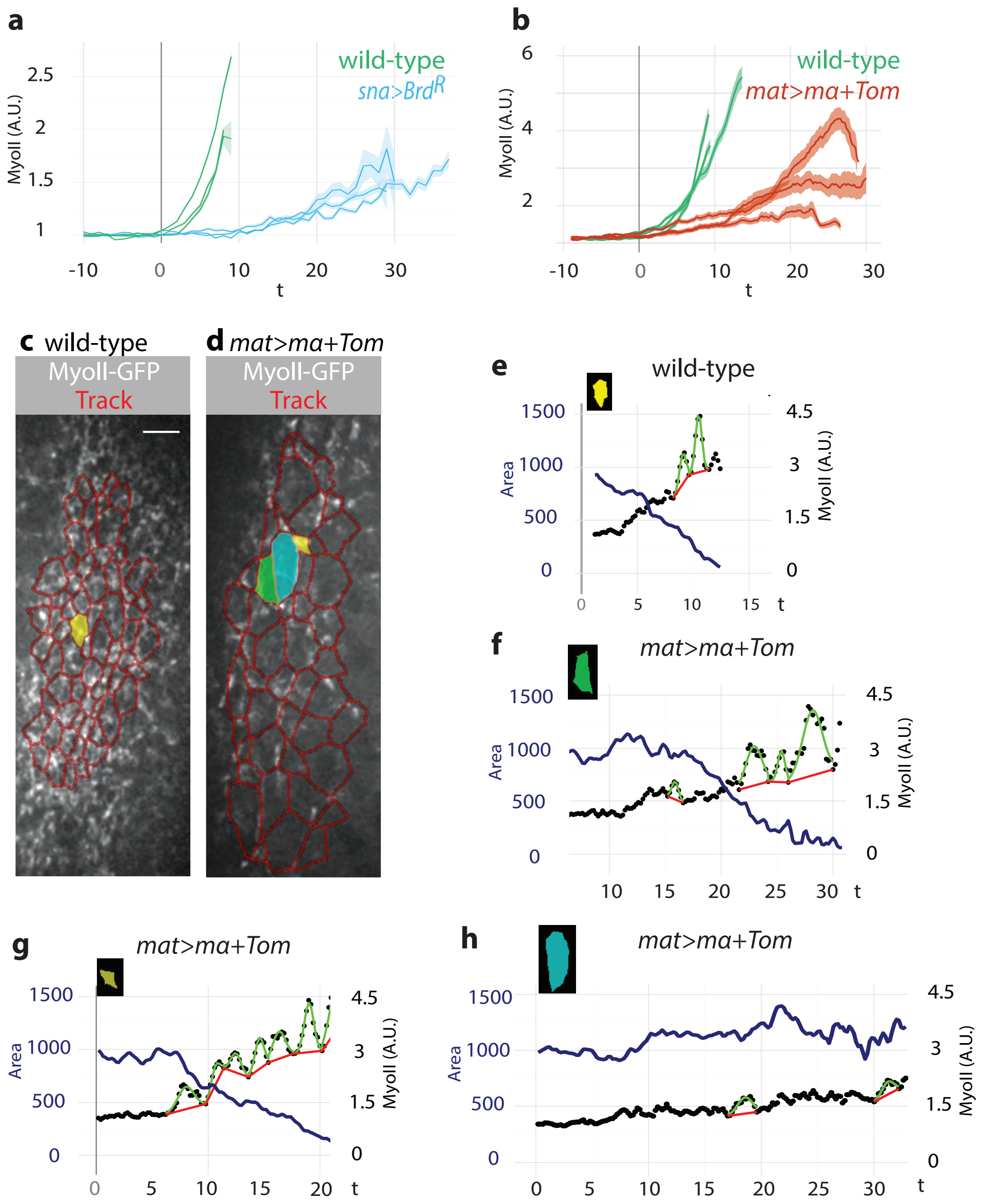

Supplementary Figure 3. *Related to Figure 2.* Ectopic Brd inhibits apical recruitment of MyoII

a) Total apical MyoII levels in ventral cells of wild-type (n=3) and *sna>Brd*^{R} embryos (n=3). Each curve corresponds to a single embryo imaged at 40x (mean values plotted in Figure 2a). Ectopic Brd slowed down the accumulation of MyoII in ventral cells.

b) Total apical MyoII levels in ventral cells of wild-type (n=3) and *mat>mα+Tom* embryos (n=3). Each curve corresponds to a single embryo imaged at 63x (mean values plotted in Figure 2b,c). For each embryo, mean Myo-GFP intensities were calculated from ~ 30-40

ventral cells. Ectopic Brd resulted in slow and delayed increase of MyoII-GFP intensity. The observed variability in MyoII levels might in part be due to embryo-to-embryo differences in transgene copy number (see Methods).

c-h) Single cell analysis of MyoII-GFP intensity (grey) in wild-type (c,e) and *mat>mα+Tom* embryos (d,f-h) revealed the existence of MyoII accumulation pulses as cells constrict (apical surface area, black in e-h). Pulse analysis is shown for one ventral cell from a wild-type embryo (color-coded in c; inset in e) and for three different cells from a *mat>mα+Tom* embryos (color-coded cells in d; insets in f-h). Distinct patterns of MyoII pulses and apical constriction were observed in these three cells: MyoII pulses varied in number, frequency and amplitude but their presence still appeared to correlate with constriction. Pulses were automatically tracked (green pulses over the red baseline), scored (pulse frequency) and MyoII intensity was measured (amplitude; see Methods).

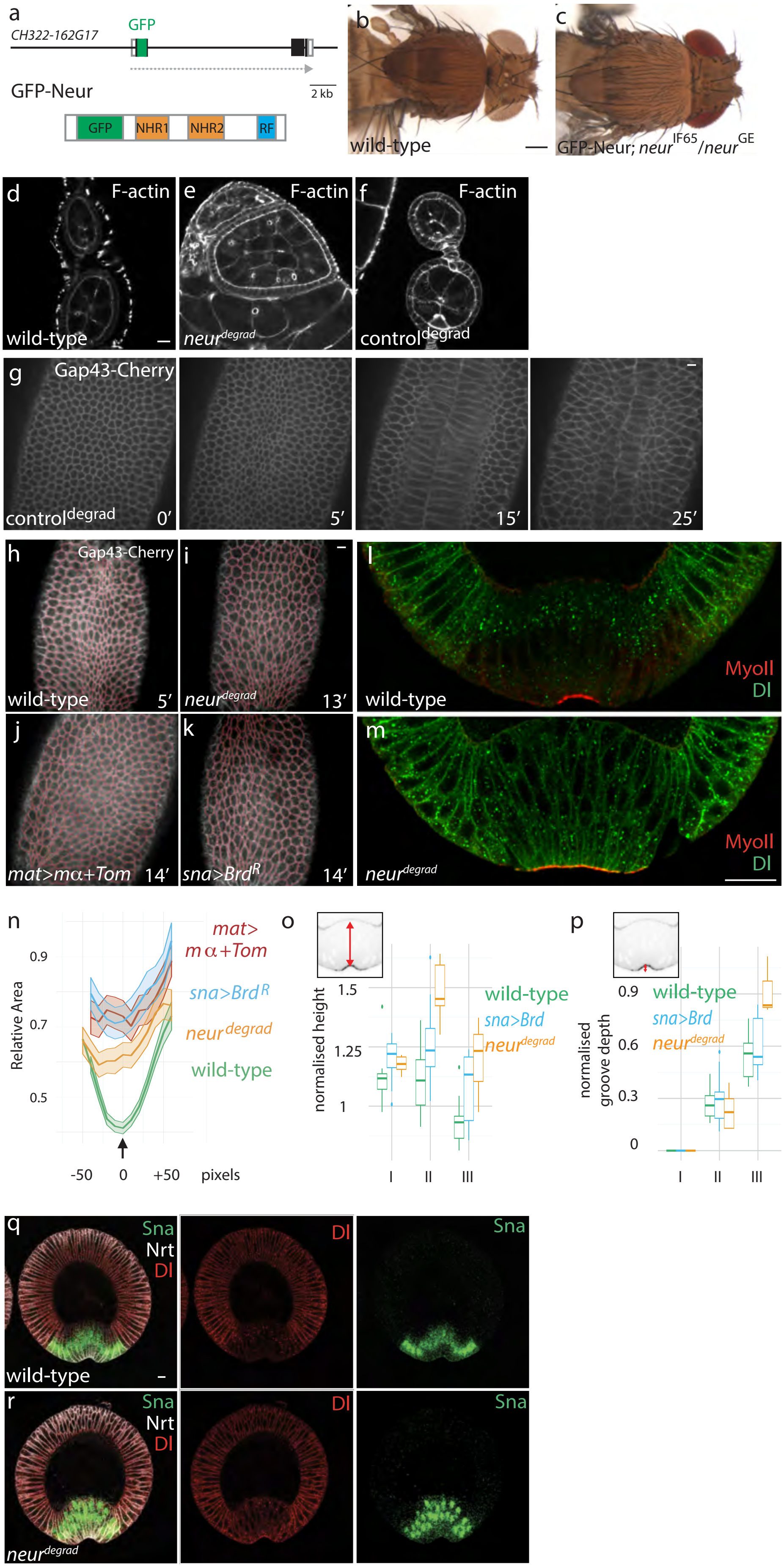
a
CH322-162G17
GFP
2 kb
GFP-Neur
GFP
NHR1
NHR2
RF
b
wild-type
c
GFP-Neur; neur IF65 /neur GE
d
F-actin
wild-type
e
F-actin
neur degrad
f
F-actin
control degrad
g
Gap43-Cherry
control degrad
0′
5′
15′
25′
h
Gap43-Cherry
wild-type
5′
i
neur degrad
13′
l
wild-type
MyoII
Dl
j
mat>mα+Tom
14′
k
sna>Brd R
14′
m
neur degrad
MyoII
Dl
n
Relative Area
0.9
0.7
0.5
-50
0
+50
pixels
mat> mα+Tom
sna>Brd R
neur degrad
wild-type
o
normalised height
1.5
1.25
1
I
II
III
wild-type
sna>Brd
neur degrad
p
normalised groove depth
0.9
0.6
0.3
0
I
II
III
wild-type
sna>Brd
neur degrad
q
Sna
Nrt
Dl
wild-type
Dl
Sna
r
Sna
Nrt
Dl
neur degrad
Dl
Sna

Supplementary Figure 4. ***Related to Figure 3.*** **Knocking-down Neur using DeGradFP**

a-c) Simplified genomic structure of the *neur* locus encoded within the 162G17 BAC. The main *neur-RA* isoform is shown (exons are boxed, ORF is in black). GFP (green) was inserted within a poorly conserved region, N-terminal to the Neur Homology Repeats (NHR1 and 2) and the catalytic Ring Finger (RF). One copy of the GFP-Neur BAC fully rescued the embryonic lethality associated with a loss of *neur* activity: trans-heterozygous $neur^{IF65}$ / $neur^{GE}$ mutant flies are viable and fertile (b,c).

d-f) F-actin staining of dissected ovaries (Phalloidin, white) showed fusion of multiple germline cysts in unfertile $neur^{degrad}$ females (e), as seen upon loss of *Dl* activity in the germ-line [1]. Such defects were not seen in wild-type and $control^{degrad}$ females (d,f). Depletion of Neur in the female germ-line using the degradFP method blocked oogenesis within a few days whereas $control^{degrad}$ females remained fertile (n=1 experiment).

g) Live imaging of a $control^{degrad}$ embryo expressing Gap43-Cherry (white). The furrow formed and invaginated properly.

h-k) Snapshot views of wild-type (h), $neur^{degrad}$ (i), *mat>m*α*+Tom* (j), and *sna>Brd*^{R} (k) embryos at mid-constriction (t= ~ 5 min in wild-type embryos and t= ~ 14 min in Neur perturbation experiments).

l,m) MyoII (red) localized apically in both wild-type (l) and $neur^{degrad}$ embryos (m). Dl (green) accumulated into endocytic vesicles in the mesoderm of wild-type embryos (l) but remained at the plasma membrane of $neur^{degrad}$ embryos (m).

n) Analysis of apical constriction along the DV axis (0 is the ventral midline, arrow) revealed a clear gradient of constriction in wild-type embryos (green; n=3; surface area values at mid-constriction as in panels h-k, i.e. when 3% of the ventral cells have constricted to reduce 5-fold their surface). By contrast, no clear minima were seen upon depletion and inhibition of Neur in the mesoderm, indicative of a more widespread distribution of apical constriction across the mesoderm ($neur^{degrad}$, orange, n=3; *mat>m*α*+Tom*, red, n=3; *sna>Brd*^{R}, blue, n=3).

o,p) Ventral cell lengthening (height, o) and furrow invagination (grove depth, p) were used to stage embryos (stage I, II and III as defined in Sweeton *et al.* [2]). Number of studied embryos: n=9, 21 and 9 for stage I, II and III wild-type embryos, respectively (same data as in Figure 3g); n = 5, 5 and 3 for $neur^{degrad}$ embryos; n= 14, 27 and 10 for *sna>Brd*^{R} embryos.

q,r) Depletion of Neur in $neur^{degrad}$ embryos (n) had no detectable effect on the expression of Snail (Sna, green) in the mesoderm (Dl, red, was used here as a control for the loss of *neur*

activity; compare with wild-type control, m). Sna-expressing cells appeared to eventually invaginate in *neur*^{degrad} embryos.

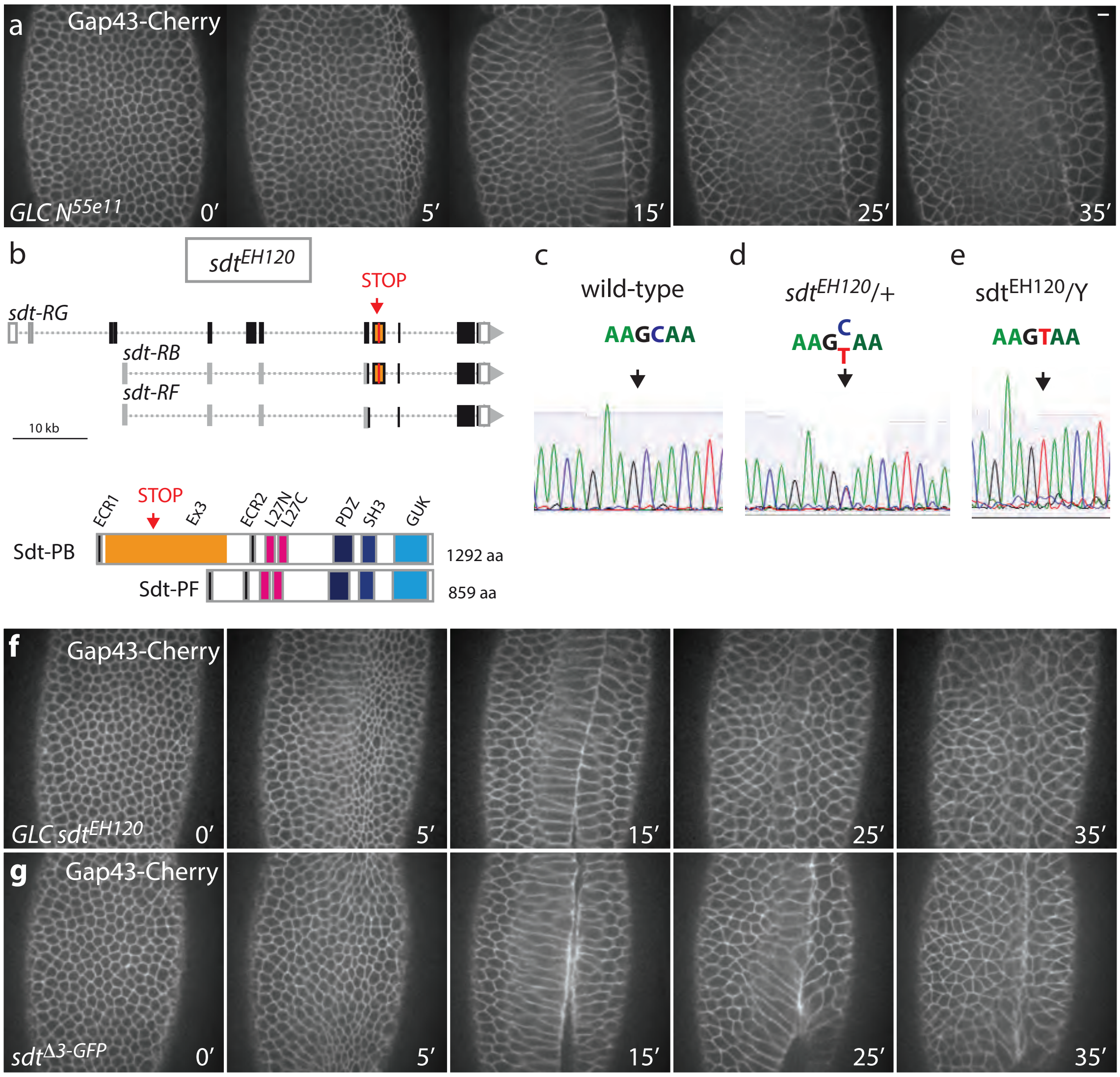

Supplementary Figure 5. *Related to Figure 3.* Stardust is not a target of Neur in the mesoderm

a) Live imaging of a *N* mutant embryos derived from a mutant germ-line clone (GLC) showing that the complete loss of *N* activity did not affect gastrulation (apical constriction was monitored using Gap43-Cherry, white).

b) Genomic organization of the *sdt* locus showing the structure of the *sdt-RB*, *-RF* and *-RG* transcript isoforms (top) as well as the domain structure of the Sdt-PB and Sdt-PF protein isoforms (bottom). The Exon3 (orange) encodes a Neur Binding Motif (NBM) and Neur targets these NBM-containing isoforms of Sdt, such as Sdt-PB, but not those lacking exon3,

such as Sdt-PF, for down-regulation in epithelia [3]. The *sdt*^{EH120} mutant allele carries a non-sense mutation within exon 3. This mutation should therefore disrupt the synthesis of all isoforms that are targeted by Neur.

c-e) Following live imaging, each *sdt*^{EH120} mutant embryos derived from GLC was individually genotyped using single-embryo PCR and sequencing of the PCR product. Hemi/homozygous mutant embryos derived from germ-line clone were unambiguously identified from heterozygous mutants.

f,g) Live imaging of a *sdt*^{EH120} GLC mutant embryo (f) and of a *sdt*$^{\Delta 3_{GFP}}$ mutant embryo (g) expressing Gap43-Cherry (white) and MyoII-GFP (not shown). The sequence of the exon3 of the *sdt* gene was replaced by the sequence of GFP using CRISPR-mediated homologous recombination to produce the *sdt*$^{\Delta 3_{GFP}}$ allele [3]. This viable mutation disrupts the regulation of Sdt by Neur. In both mutants, no defect in furrow formation and invagination could be observed. This showed that the Sdt isoforms interacting with and regulated by Neur are not required for gastrulation.

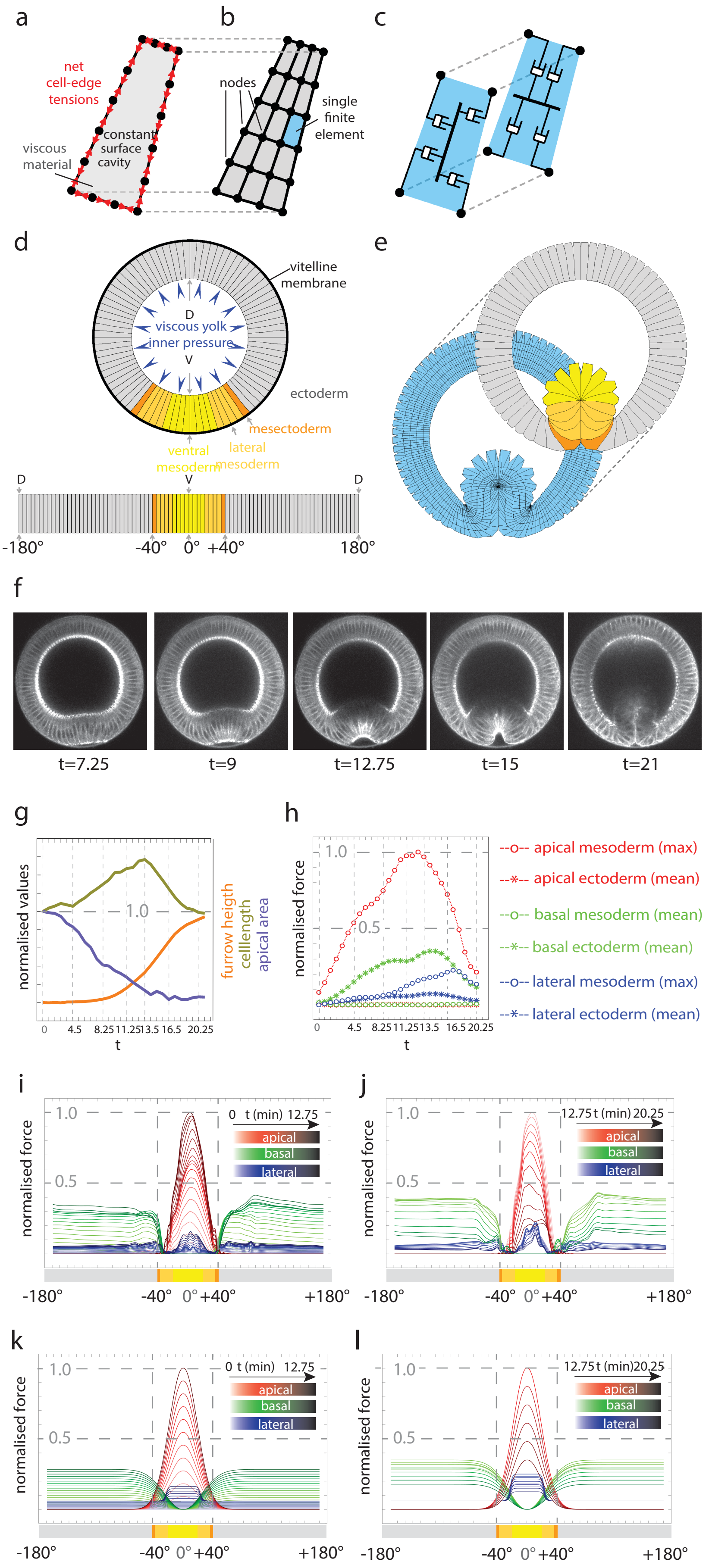
a
b
c
net
cell-edge
tensions
nodes
single
finite
element
constant
surface
cavity
viscous
material
d
e
vitelline
membrane
D
viscous yolk
inner pressure
V
ectoderm
mesectoderm
lateral
mesoderm
ventral
mesoderm
D
V
D
-180°
-40°
0°
+40°
180°
f
t=7.25
t=9
t=12.75
t=15
t=21
g
h
normalised values
1.0
furrow heigth
celllength
apical area
0
4.5
8.25
11.25
13.5
16.5
20.25
t
normalised force
1.0
0.5
--o-- apical mesoderm (max)
--*-- apical ectoderm (mean)
--o-- basal mesoderm (mean)
--*-- basal ectoderm (mean)
--o-- lateral mesoderm (max)
--*-- lateral ectoderm (mean)
i
j
k
l
normalised force
1.0
0.5
0 t (min) 12.75
12.75 t (min) 20.25
apical
basal
lateral
-180°
-40°
0°
+40°
+180°

Supplementary Figure 6. ***Related to Figure 4.*** **Multi-scale 2D model and force distributions obtained through VFM**

a) Model of a single epithelial cell in which forces (red) act along the cell cortex and on the incompressible viscous cytoplasm to emulate the net contribution of all cellular force fields.

b) Each cell is subdivided into 15 finite elements (blue) that locally emulate the viscous response of the cell [4]. These are used to calculate the deformations generated by cortical forces and viscous cell components during each time step of the simulation according to the mathematical formulation described earlier [4, 5].

c) Dashpots used to model each viscous finite element that locally emulates the cellular interior. Two sets of dashpots orthogonal to each other are overlaid and joint at nodes [5].

d) The reference configuration of the finite element model matching the wild type embryo at the cellular level is shown as a cross-section (top). The corresponding one-dimensional topology used in Figure 4 is shown below.

e) Epithelial geometry of a wild-type embryo at the end of furrow invagination (top layer) and corresponding underlying breakdown in finite elements at the subcellular level (bottom layer).

f) Multiphoton cross-section views of a wild-type embryo expressing MyoII-GFP during ventral furrow formation (taken from Brodland *et al.* [6]).

g) Temporal evolution of the measured height of the furrow (furrow invagination, orange; arbitrary unit), apical constriction (central mesoderm area, blue; arbitrary unit) and ventral cell lengthening (central mesoderm area, green; arbitrary unit) in the wild-type embryo shown in (f).

h) Temporal profile of force distributions determined by VFM in the wild-type embryo shown in (f). This data set was used as a template for the temporal profile used for simulations (see Figure 4d).

i,j) Temporal-spatial profiles of force distributions obtained by VFM in the wild-type embryo shown in (f). Temporal evolution is shown in color gradients (t ranges from 0 to 13 min in panel I, and from 13 to 20 min in panel j). Spatial profiles are represented over a one-dimensional epithelium (color code: ectoderm, grey; mesectoderm, dark orange; lateral mesoderm, light orange; central mesoderm, yellow; dorsal is at ±180°, ventral at 0°). Apical forces (red) are maximal in ventral cells whereas basal forces (green) are maximal in the ectoderm.

k,l) Temporal-spatial profiles of force distributions that were derived from the VFM data and used for simulations of the wild-type embryo (see Figure 4a,d).

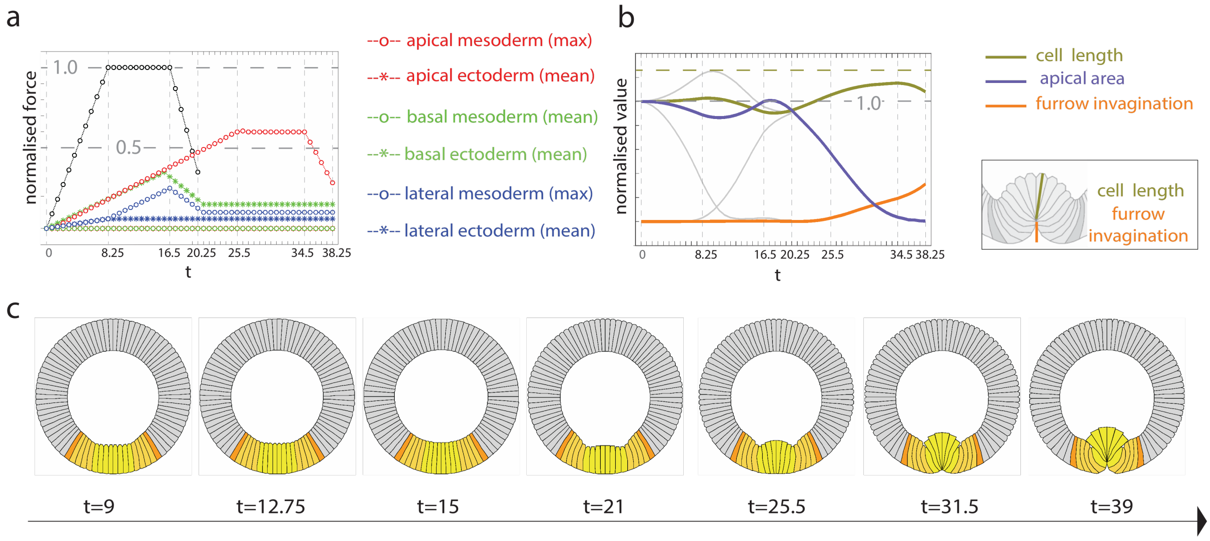

Supplementary Figure 7. ***Related to Figure 4.*** **Reduced and delayed apical forces do not account for ventral cell lengthening**

a) Time evolution of force distribution was modified so that apical forces (red) took longer to reach a maximum value weaker than their wild-type counterparts (black; see Figure 4d). All other force profiles remained unchanged.

b) Time trends of apical constriction (blue), furrow invagination (orange) and ventral cell lengthening (green) associated with the force profiles shown in a (wild-type counterparts are shown in grey; see Figure 4g). Note that ventral cell elongated less than in the simulation of wild-type embryo (max elongation value indicated as a dotted green line for comparison)

c) Selected *in silico* views of the corresponding simulation. Note that ventral cells adopted unusual shape that were not observed upon inhibition/depletion of Neur (see t=25.5).

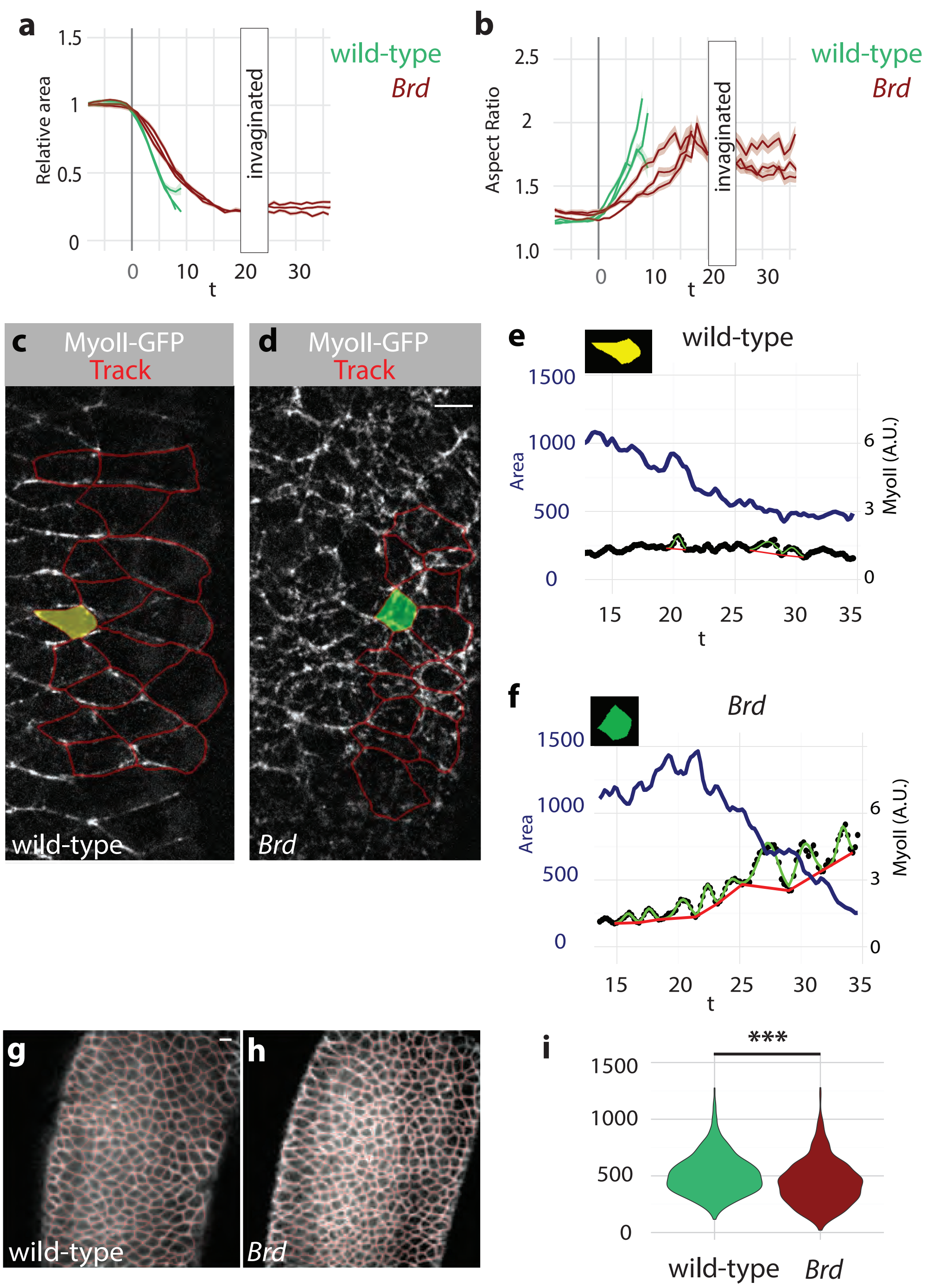

Supplementary Figure 8. ***Related to Figure 5.*** **Apical constriction and MyoII dynamics in *Brd* mutant embryos**

a) Apical constriction (mean surface values of constricting ventral cells plotted over time) appeared to be slightly slower in *Brd* mutant embryos (brown, n=3) compared to wild-type controls (green, n=3; same data as in Supplementary Figure 1d).

b) As ventral cells constrict, their surface area become anisotropic (as seen by measuring their aspect ratio) in both wild-type (green, n=3) and *Brd* mutant embryos (brown, n=3).

Thus, increased contractility in the ectoderm correlated with decreased anisotropy in ventral cells in *Brd* mutant embryos.

c-f) Single cell analysis of MyoII-GFP intensity (grey) in wild-type (c) and *Brd* embryos (d) showed that a higher number of strong MyoII pulses could be measured in the ectoderm of *Brd* mutant embryos relative to control embryos (e,f). Pulse analysis is shown for a representative lateral cell from a wild-type embryo (color-coded in c; inset in e) and a *Brd* mutant embryos (color-coded in d; inset in f). Stronger pulses were detected in *Brd* mutants. These appeared to correlate with apical constriction.

g-i) Snapshot views of the lateral ectoderm of wild-type (g) and *Brd* (h) mutant embryos soon after furrow invagination (Gap43-Cherry, white and tracking, red). Consistent with increased apical contractility, the apical area of lateral ectoderm cells was found to be reduced in *Brd* mutant embryos (793 cells, 3 embryos) relative to control embryos (904 cells, 4 embryos; distributions shown as mirrored density, i.e. violin, plots in i, $p=3x10^{-6}$).

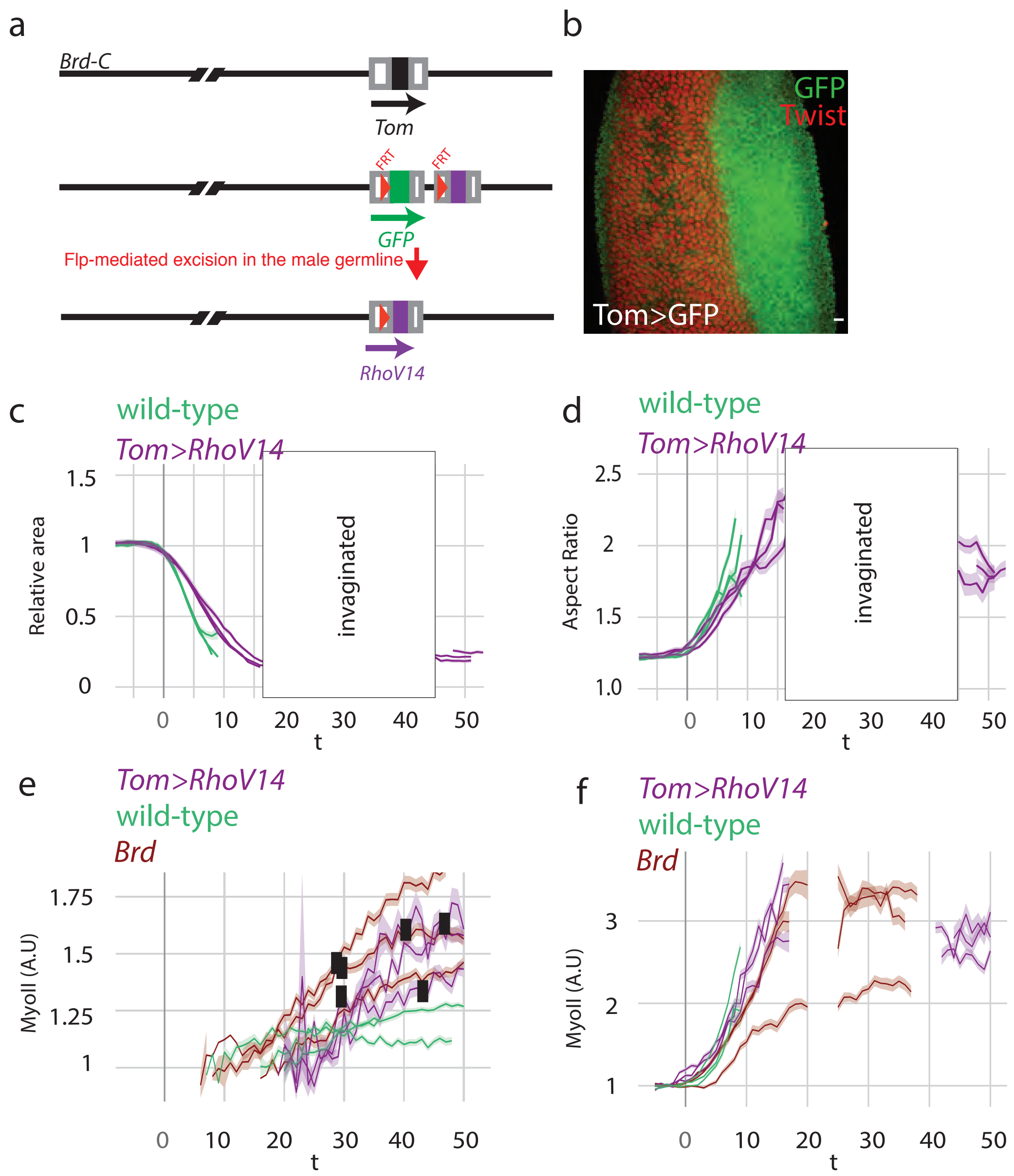

Supplementary Figure 9. ***Related to Figure 6.*** **Conditional increase in Rho signaling in the ectoderm**

a,b) Strategy to express RhoV14 in the ectoderm. The *Tom* ORF was replaced by a FRT-GFP-stop-FRT-RhoV14 construct (a) to produce a *Tom>GFP* transgene. This transgene directed the expression of GFP (green; Twist, red) in the ectoderm (b; n= 2 experiments). Flp-mediated excision of the FRT-GFP-stop-FRT cassette produced a *Tom>RhoV14* transgene expressing RhoV14 in the ectoderm. Genotyping, i.e. molecular analysis of the recombination event, was achieved after imaging by single-embryo PCR analysis of the transgene.

c) Analysis of furrow invagination in *Tom>RhoV14* embryos. Apical constriction (mean surface values of constricting ventral cells plotted over time) appeared to be slightly slower in *Tom>RhoV14* embryos (purple, n=3) compared to wild-type controls (green, n=3; same data as in Supplementary Figure 1d). Increased Rho activity in the ectoderm mimicked the effect of loss of *Brd* activity on the speed of invagination (Supplementary Figure 8a).
d) Increasing Rho signaling activity in the ectoderm (in *Tom>RhoV14* embryos; purple, n=3) led to decreased anisotropy in ventral cells (compare with wild-type embryos, green; n=3). Again, this effect is similar to the one seen in *Brd* mutants (Supplementary Figure 8b).
e,f) MyoII-GFP intensity was measured in the ventral ectoderm (e) and mesoderm (f) of wild-type (green, n=3), *Brd* mutant (brown, n=3) and *Tom>RhoV14* (purple, n=3) embryos. Intensity values were plotted over time for individual embryos (mean curves are shown in Figure 6c). For each embryo, the onset of furrow unfolding is indicated with a black stamp (e). In the mesoderm, MyoII levels increased similarly in all genotypes during apical constriction (from t=0 to 10) but ventral cells eventually reached higher levels of MyoII accumulation in *Brd* and *Tom>RhoV14* embryos than wild-type cells (f). One possible interpretation is that tension builds up in ventral cells as the furrow fails to fully invaginate.

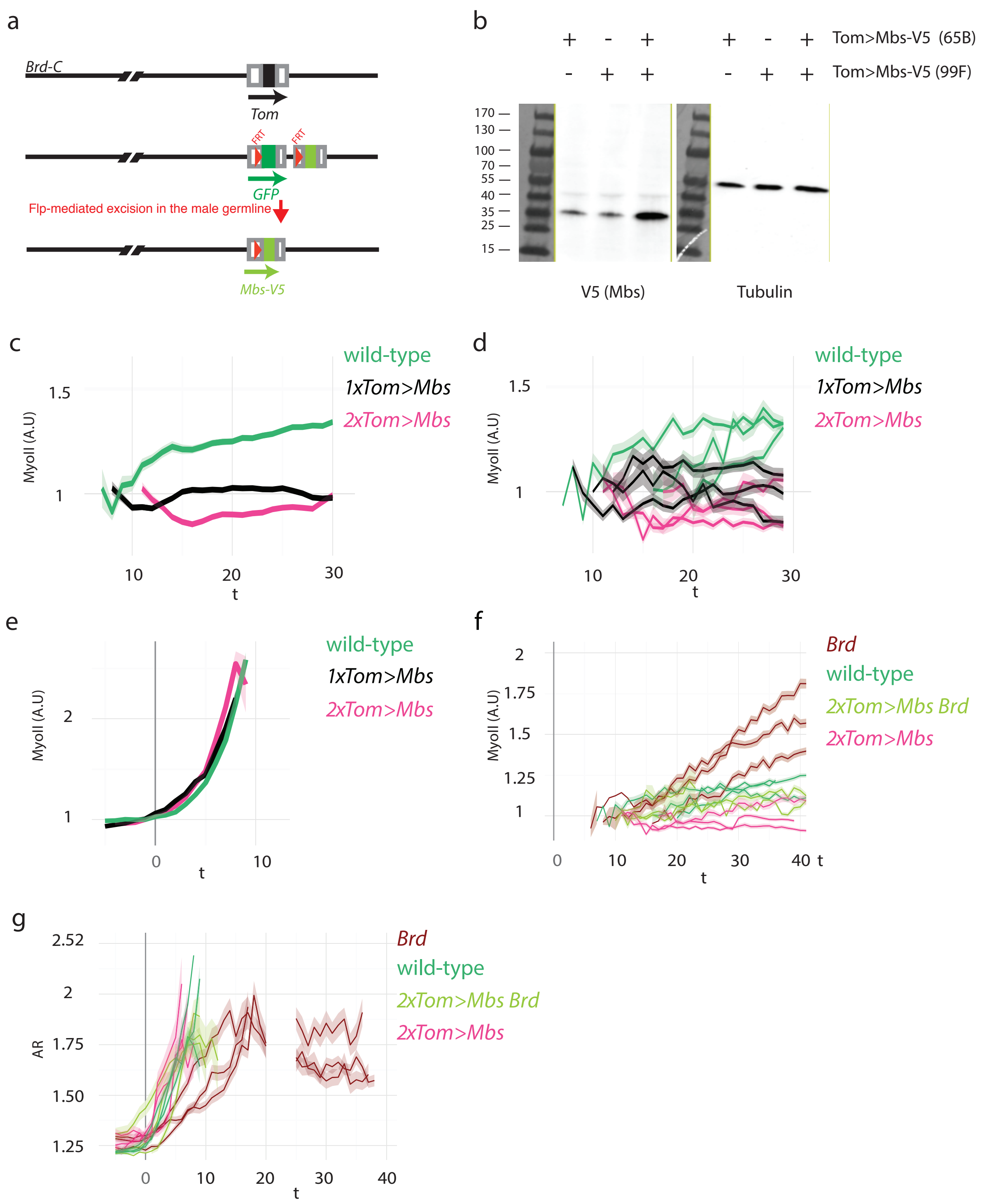

Supplementary Figure 10. ***Related to Figure 7.*** **Conditional decrease in MyoII activity in the ectoderm**

a) Strategy to express an active V5-tagged form of Mbs, MbsN300-V5. Conditional expression of Mbs was achieved using the regulatory sequences of the *Tom* gene together with a conditional stop cassette. Genotyping was performed following imaging using single-embryo PCR analysis of the transgene.

b) Two copies of the Tom>Mbs-V5 transgene were used to achieve high expression levels as determined by single-embryo anti-V5 Western Blot analysis (+ denotes the presence of the flipped-out version of the corresponding transgene; the anti-Tubulin signal was used as a loading control; the position of molecular weight markers is indicated). The full scan of the western blot with the molecular weights is shown (n=1 experiment).

c,d) MyoII-GFP intensity was measured in ventral ectodermal cells of wild-type (green, n=3; individual embryos are shown in d, mean values in c), *1x Tom>Mbs* (black, n=3), and *2x Tom>Mbs* (pink, n=3) embryos. Mbs decreased MyoII levels in the ectoderm. An earlier decrease was seen with 2 copies of Mbs.

e) Expression of Mbs in the ectoderm of *1xTom>Mbs* (black, n=3) and *2xTom>Mbs* (pink, n=3) embryos did not affect the increase in MyoII levels in ventral cells (wild-type, green). Mean values are shown.

f) The expression of Mbs in the ectoderm of *Brd* mutant embryos strongly limited the accumulation MyoII-GFP in these mutant embryos. MyoII-GFP was measured in ventral ectodermal cells of wild-type (green, n=3; same embryos as in Supplementary Figure 9e), *Brd* (brown, n=3; same embryos as in Supplementary Figure 9e), *Brd 2xTom>Mbs* (light green, n=3), and *2xTom>Mbs* embryos (pink, n=3; as plotted in c').

g) The expression of Mbs also restored anisotropy of the constricting ventral cells in *Brd* mutant embryos (aspect ratio, AR; see also Supplementary Figure 8b), further indicating that contractility in the ectoderm influence apical constriction in the mesoderm.

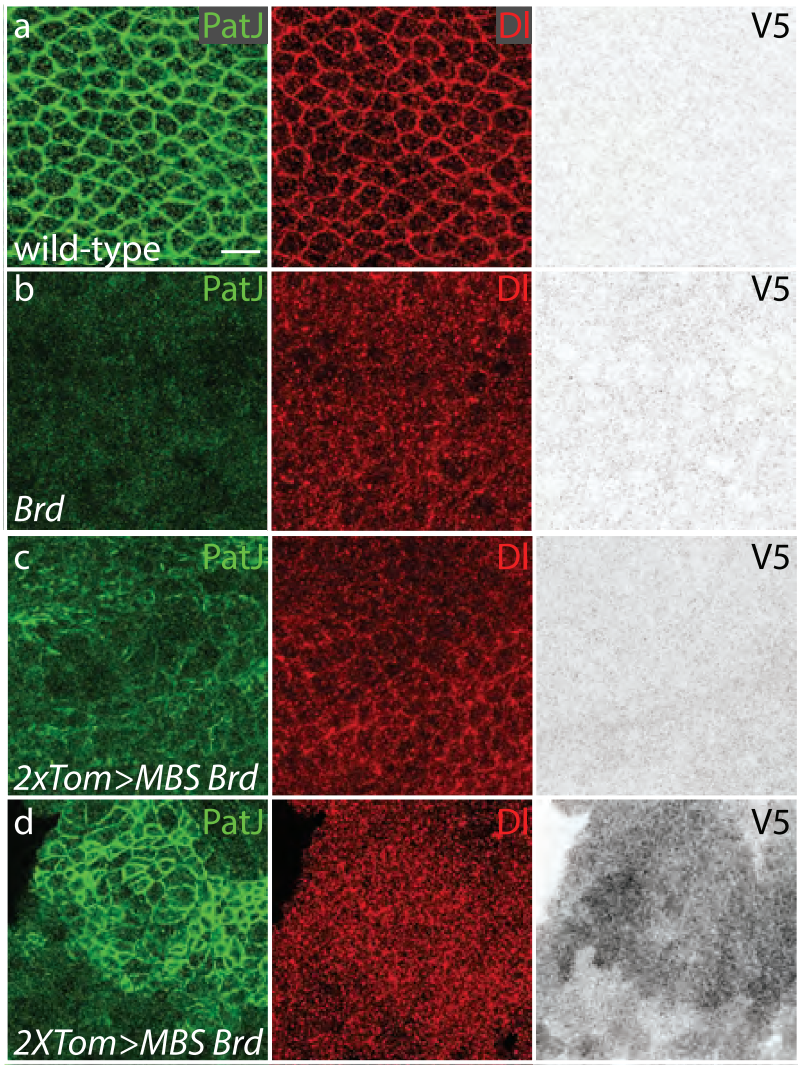

Supplementary Figure 11. ***Related to Figure 7.*** **Ectopic Mbs partially suppressed the *Brd* epithelium integrity phenotype**

Analysis of epithelium integrity in early embryos (stage 8, a-c) confirmed that loss of *Brd* resulted in a loss of epithelial polarity (b; wild-type control, a), as revealed by the loss of Patj (green; Neur-dependent Dl endocytosis is also increased in *Brd* mutant embryos, leading to a loss of membrane Dl staining, red) and showed that ectopic expression of Mbs (V5, black) was not sufficient to suppress these defects in Dl and Patj localization in early embryos (c), consistent with Neur regulating Dl and Patj (via Sdt) in a largely MyoII-independent manner. Neverthless, epithelium integrity was partially restored in the dorsal ectoderm of stage 12 *Brd 2xTom>Mbs* embryos (d). This suggests that the *Brd*-dependent increase in apical contractility may contribute to the loss of epithelial polarity seen in *Brd* mutant embryos, particularly in the dorsal ectoderm that does not undergo major morphogenesis events, such as germ-band extension and neuroblast delamination.

Supplementary references

1. Lopez-Schier, H. & Johnston, D.S. Delta signaling from the germ line controls the proliferation and differentiation of the somatic follicle cells during Drosophila oogenesis. *Developmental cell* **15**, 1393-1405 (2001).
2. Sweeton, D., Parks, S., Costa, M. & Wieschaus, E. Gastrulation in Drosophila: the formation of the ventral furrow and posterior midgut invaginations. *Development* **112**, 775-789 (1991).
3. Perez-Mockus, G., Roca, V., Mazouni, K. & Schweisguth, F. Neuralized regulates Crumbs endocytosis and epithelium morphogenesis via specific Stardust isoforms. *J Cell Biol* **216**, 1405-1420 (2017).
4. Conte, V. *et al.* A biomechanical analysis of ventral furrow formation in the Drosophila melanogaster embryo. *PLoS One* **7**, e34473 (2012).
5. Brodland, G.W., Viens, D. & Veldhuis, J.H. A new cell-based FE model for the mechanics of embryonic epithelia. *Comput Methods Biomech Biomed Engin* **10**, 121-128 (2007).
6. Brodland, G.W. *et al.* Video force microscopy reveals the mechanics of ventral furrow invagination in Drosophila. *Proc Natl Acad Sci U S A* **107**, 22111-22116 (2010).
