# Supplementary material for: Spatial regulation of contractility by Neuralized and Bearded during furrow invagination in Drosophila
Source: Nat Commun. 2017 Nov 17;8:1594. doi: 10.1038/s41467-017-01482-8 (PMC5693868; doi:10.1038/s41467-017-01482-8)
Supplement: Supplementary file 3 — Description of Additional Supplementary Files [file 41467_2017_1482_MOESM3_ESM.docx]

**Description of Additional Supplementary Files**

**File name:** Supplementary Movie 1

**Description:** Live imaging of wild-type (left), *mat>mα+Tom* (middle) and *sna>Brd^R^* (right) embryos expressing a membrane marker (Gap43-Cherry). Central mesoderm cells (blue) were segmented and tracked (red). Inhibition of Neur by proteins of the Brd family perturbs collective apical constriction and delays furrow invagination. In this and all other movies, time is in min. and the onset of apical constriction in individual cells defines t=0.

**File name:** Supplementary Movie 2

**Description:** Live imaging of wild-type (left) and *mat>mα+Tom* (right) embryos expressing MyoII-GFP (top panels; green in bottom panels) and a membrane marker (Gap43-Cherry, red). Over-expression of the Brd proteins Tom and m*α* leads to reduced apical MyoII levels and decreased contractility.

**File name:** Supplementary Movie 3

**Description:** Live imaging of a *neur^degrad^* embryo expressing a membrane marker (Gap43-Cherry). Central mesoderm cells (blue) were segmented and tracked (red). Depletion of Neur affects collective apical constriction and slows down furrow invagination.

**File name:** Supplementary Movie 4

**Description:** Ventral furrow invagination of wild-type and *neur* *in silico* embryos. See Figure 4 for the spatial-temporal distribution of forces used here.

**File name:** Supplementary Movie 5

**Description:** Live imaging of a *Brd* mutant embryo expressing a membrane marker (Gap43-Cherry). Central mesoderm cells (blue) were segmented and tracked (red). Loss of *Brd* activity results in furrow unfolding.

**File name:** Supplementary Movie 6

**Description:** Ventral furrow invagination (and unfolding) of wild-type and *Brd* *in silico* embryos. See Figure 4 for the spatial-temporal distribution of forces used here.

**File name:** Supplementary Movie 7

**Description:** Live imaging of wild-type (left) and *Brd* mutant (right) embryos expressing MyoII-GFP (top panels; green in bottom panels) and a membrane marker (Gap43-Cherry, red). Increased apical MyoII levels and formation of contractile apical-medial MyoII meshworks in the ectoderm correlated with furrow unfolding in *Brd* mutant embryos.

**File name:** Supplementary Movie 8

**Description:** Live imaging of wild-type (left), *Brd* mutant (middle) and *Tom>RhoV14* (right) embryos expressing MyoII-GFP (top panels; green in bottom panels) and a membrane marker (Gap43-Cherry, red). Increased apical MyoII levels and formation of contractile apical-medial MyoII meshworks in the ectoderm correlated with furrow unfolding in *Brd* mutant embryos. Note that MyoII increased mostly at junctions in *Tom>RhoV14* embryos. Note also the strong MyoII signal at the medial-apical cortex of ventral cells, suggestive of constricted meshworks, as the furrow unfolds in *Brd* mutant embryos.

**File name:** Supplementary Movie 9

**Description:** Live imaging of a *Tom>RhoV14* embryo expressing a membrane marker (Gap43-Cherry). Central mesoderm cells (blue) were segmented and tracked (red). Increased Rho activity in the ectoderm results in furrow unfolding.

**File name:** Supplementary Movie 10

**Description:** Live imaging of *Brd* mutant embryos with two flipped-out copies of the *Tom>Mbs* transgene and expressing a membrane marker (Gap43-Cherry). Expression in the ectoderm of a MyoII phosphatase suppressed the furrow unfolding phenotype seen in *Brd* mutant embryos.

**File name:** Supplementary Movie 11

**Description:** Live imaging of *2xTom>Mbs* embryo and expressing a MyoII-GFP (top, white and bottom, green) and Gap43-Cherry (red). Expression in the ectoderm of a MyoII phosphatase suppressed MyoII accumulation (compare with Movie 8).
